# Autoimmune Congenital Heart Block: A Review of Biomarkers and Management of Pregnancy

**DOI:** 10.3389/fped.2020.607515

**Published:** 2020-12-22

**Authors:** Sara De Carolis, Cristina Garufi, Ester Garufi, Maria Pia De Carolis, Angela Botta, Sara Tabacco, Silvia Salvi

**Affiliations:** ^1^Fondazione Policlinico Universitario “A. Gemelli” IRCCS, Department of Obstetrics, Gynaecology and Pediatrics, Rome, Italy; ^2^Dipartimento di Scienze Cliniche, Internistiche, Anestesiologiche e Cardiovascolari, UOC Reumatologia, Sapienza University of Rome, Rome, Italy; ^3^Medical School, University of Florence, Florence, Italy; ^4^Department of Gynecological Sciences, Sapienza University of Rome, Rome, Italy

**Keywords:** precision medicine, hydroxycloroquine, PR interval, type I Interferon, anti-Ro/SSA antibodies, autoimmune congenital heart block

## Abstract

Autoimmune Congenital Heart Block (CHB) is an immune-mediated disease due to transplacental passage of circulating anti-Ro/SSA and anti-La/SSB autoantibodies. It occurs in 2% of anti-Ro/SSA-exposed pregnancies, and recurrence rate is nine times higher in subsequent pregnancies. Aim of this review is to identify biomarkers of CHB and treatment strategies. The Ro-system is constituted by two polypeptides targeted by the anti-Ro52 and anti-Ro60 autoantibodies. The central portion of Ro52 (p200), more than the full amino-acid sequence of Ro-52, is recognized to be the fine specificity of anti-Ro associated to the highest risk of cardiac damage. If anti-p200 antibody should be tested, as biomarker of CHB, over standard commercial ELISAs is still debated. Recent studies indicate that type I-Interferon (IFN) can activate fibroblasts in fetal heart. In the mother the anti-Ro/La antibodies activate the type I IFN-signature, and maternal IFN-regulated genes correlate with a similar neonatal IFN-gene expression. Evaluation of maternal IFN-signature could be used as novel biomarker of CHB. The measurement of “mechanical” PR interval with weekly fetal echocardiogram (ECHO) from 16 to at least 24 weeks of gestation is strongly recommended for CHB prenatal diagnosis. However, ECHO screening presents some limitations due to difficult identification of first-degree block and possible occurrence of a complete block from a normal rhythm in few days. Maternal administration of Hydroxychloroquine from the tenth week of gestation, modulating toll-like receptor and autoantibody-dependent type I IFN activation on the fetus, has an important role in preventing CHB in pregnant women with high risk for recurrent CHB.

## Highlights

- Maternal antibodies profile can identify women at higher risk for delivering child with CHB: combination of anti-Ro52 and anti-Ro60 antibodies, especially at high level could recognize women at risk for having an infant with CHB.
- Evaluation of maternal Interferon (IFN) score could be used as novel biomarker of CHB.
- The PR interval measurement by ECHOs is a valuable tool for identification of early and potentially reversible conduction abnormalities in fetuses at risk for more advanced and permanent forms of CHB. It is appropriate to perform weekly monitoring at least between 16 and 24 weeks.
- A “biomarker” of reversible injury, such as the PR interval prolongation above 150 ms, is a useful tool also to begin an early treatment.
- As regards the pharmacological prophylaxis of CHB during pregnancy, Hydroxychloroquine, when initiated early in pregnancy 400 mg daily, resulted in a risk reduction of >50% in the recurrence of CHB. Its use is recommended starting from the 10th weeks' gestation because its long half-life of 2 months in women with high risk of recurrence.

## Introduction

Autoimmune congenital heart block (CHB) is part of Neonatal Lupus Syndrome and is a model of passively acquired autoimmunity disease, caused by transplacental passage of circulating anti-Ro/SSA and anti-La/SSB autoantibodies to fetal conduction cardiac tissue (1, 2). Nevertheless, considering the low CHB penetrance in antibodies-exposed pregnancies, other factors can influence the clinical manifestation. CHB incidence is 2% in anti-Ro/SSA positive women, 3% in presence of both anti-Ro/SSA and anti-La/SSB, but it results higher in patients suffering active disease and/or having elevated antibody levels (3). The recurrence in cases with previous affected child is almost 16–18% (4–6): it is nearly nine times higher in comparison to that of antibodies-exposed primigravidae despite the persistence of maternal antibodies, indicating that other maternal, fetal, and environmental factors may influence the immune response and the fibrotic reaction in myocardial tissue.

The progression from normal sinus rhythm to CHB can occurs in a few days, as observed by monitoring of fetal echocardiography (ECHO) (7).

CHB has significant morbidity and mortality both before and after birth (8). The mortality rate ranges from 12 to 43%. Most of surviving affected infants need pacing before adulthood. A rate of live birth of 80% was detected by the Italian Registry of autoimmune CHB, with two-thirds of the children who needed pacemaker implantation after birth (9).

To date, diagnosis of CHB is challenging and the best management is still questioned.

The use of dexamethasone or intravenous immunoglobulin (IVIG) in order to reverse second-degree atrioventricular block (AVB) in utero is reported to be successful in 20–25% of cases (10), but even when improvement occurs, regression back to CHB still occurs in about one-half of infants (3). Third-degree AVB or complete CHB (CCHB) is considered permanent (11) and, as demonstrated in a recently meta-analysis, no medical treatment (fluorinated steroids) is superior to other one (alpha-sympathomimetic drug, IVIG and plasma exchange) (12).

Since CCHB is permanent, different studies have tried to predict those fetuses who will develop conduction abnormalities by identifying a possible biomarker for incomplete CHB.

The purpose of this review is to analyse the literature devoted to identify some good “biomarkers,” defined as something that can be measured accurately and reproducibly to early identify fetuses at higher risk for CHB. Our review will consider which is the best management during pregnancies in women at risk for having fetuses with CHB. Finally, the maternal pharmacological prophylaxis of CHB currently validated will be discussed.

### Anti-Ro/SSA Antibody: Pathogenetic Role and Possible Biomarker

The molecular mechanisms by which anti-Ro/SSA antibody can cause heart injury are not yet fully understood. Two main hypotheses have been proposed: the first one is due to inflammatory-response, and the second to molecular mimicry. The inflammatory response is due to production of immune-complexes caused by apoptotic cells exposing self-antigens that interact with maternal anti-Ro/SSA antibody, determining a remodeling of cardiomyocytes during myocardial formation (13). On the other hand, or in combination with the first mechanism, the molecular mimicry between the autoantibodies and surface antigen of the sarcolemma, in particular the L-type Ca channels, could cause dysregulation of calcium homeostasis and consequently determine toxic effect to cardiomyocytes (14).

Ro/SSA is a ribonucleoprotein constituted by two polypeptides, the 52 kDa and the 60 kDa: those peptides are targeted by the anti-52Ro and anti-60Ro autoantibodies (15).

Most of the evidence suggests the major role of anti-Ro52 in the initial damage of CHB (16). The type and the titer of the autoantibodies are also associated with tissue damage, because a significant link between anti-Ro/SSA levels and development of CHB is reported (17).

The central portion (amino acids 200–239) of Ro52, also known as p200, more than the full amino acid sequence of Ro-52, is recognized to be the fine specificity of anti-Ro associated to the highest risk of cardiac damage, considering its high prevalence in patients having affected child (18).

However, the role of anti-p200 antibodies on the genesis of CHB is still debated, as well as its possible use as biomarker of cardiac damage (19).

Tonello et al. reported 100% prevalence of both anti-Ro52 and anti-Ro60 antibodies in mothers with affected fetus, and a 93.8% prevalence of anti-p200. Moreover, the anti-p200, anti-Ro52, and anti-Ro60 antibodies combination is significantly more frequent in mothers with affected fetuses in comparison to the mothers with unaffected fetuses (*p* = 0.03), even if no relationship was observed between the anti-p200 titers and the CHB severity (20). Because anti-p200, anti-Ro52, and anti-Ro60 antibodies, especially at high level, seem to identify pregnancies at higher risk for CHB, their finding can help the clinicians in identifying cases at major risk (20). On the opposite, a different profile of risk emerged by the study of Reed et al. (21). They reported that the reactivity to p200 did not add an increased risk to conduction abnormalities over the Ro52 or Ro60 antibodies. Anti-Ro52 and p200 seemed to have low specificity (50%), anti-p200 antibodies had the least sensitivity but had the lowest probability to be false-positive in mothers of unaffected child (21).

In our opinion, on the basis of the different results of the aforementioned studies regarding anti-p200, testing only for reactivity to p200 results not sufficient to recognize mothers with high risk of CHB. Moreover, anti-p200 antibodies are still not available in commercial kits, so routinely screening for these antibodies would not be easily achievable.

### Type I IFN: Pathogenetic Role and Possible Biomarker

Heart immunohistochemistry from autopsies of fetuses died of CHB showed fibrosis, calcification, and presence of macrophages/multinucleated giant cells in the tissue of the atrioventricular (AV) node (22). Macrophages, particularly the sialic acid-binding Ig-like lecithin 1 (SIGLEC-1) positive macrophages, were the most prevalent cells in the cardiac lesions (23). These cells can increase the inflammatory response by the enrolment of other mononuclear cells.

Since the expression of SIGLEC-1 is upregulated by type I IFN, recently the role of IFN and IFN-stimulated genes in the pathogenesis of CHB have been investigated. Type I IFN upregulates Ro52 and stimulates apoptosis, so that it results likely to be a key cytokine in CHB development (24). A significantly higher expression of SIGLEC1 and IFN-α was demonstrated in mothers of children with CHB in comparison to mothers with unaffected children (25).

The recent study of Hedlung et al. (26) showed an amplified expression of INF-regulates genes and high circulating INF-alfa levels either in anti-Ro/La-exposed mothers and their neonates. A significantly higher IFN gene expression in peripheral blood mononuclear cells was detected in anti-Ro/La-exposed neonates, as well as in the anti-Ro positive mothers, in comparison with the gene expression detected in neonates born from healthy mothers. In addition, a correlation between IFN scores in mothers and their offspring has been identified, whereby evaluation of maternal IFN score could be used as novel biomarker for CHB risk (26).

### Prenatal Diagnosis: Fetal Echocardiograms (ECHOs) for PR Interval Measurement

The management of anti-Ro/La-exposed pregnancies remains heterogeneous across different centers. Although the use of ECHO screening vs. heart rate monitoring is differently applied, fetal echocardiograms (ECHOs) for PR interval measurement is the most useful low-invasive means for surveillance of fetuses at risk of CHB.

The antenatal prediction of CHB is possible by the evaluation of the “mechanical” PR interval with serial fetal ECHOs, beginning at the 16^th^-18^th^ gestational week, since CHB development usually occurs between 18 and 24 weeks of gestation (1, 27–29).

Different techniques are reported to measure the fetal mechanical PR interval. The majority of them are ultrasound-derived and denote the mechanical substitutes of electrical events. Three methods to calculate the AV interval are described, as follows (30):

1. MV-Ao: the AV interval was measured from the intersection of the mitral E and A waves to the onset of the ventricular ejection wave in the aortic (Ao) outflow.
2. MV: this time interval starts with the same event, but ends at the closure of the mitral wave (MV).
3. SVC-Ao: this time interval was measured from the beginning of the retrograde venous a-wave in the superior vena cava (SVC) to the beginning of the Ao ejection wave.”

Glickstein et al. by using MV-Ao technique firstly reported that the mechanical PR with the fetal pulsed Doppler ECHO was technically feasible. Moreover, this technique resulted independent of gestational age and showed a good relationship with electrical neonatal PR-interval (31). These results were subsequently validated by a prospective fetal ECHO study (32).

In normal fetuses the mechanical PR interval was seen to range from 90 to 150 ms, independently of gestational age and heart rate (33). Subsequently, a prospective study on normal fetuses established normal values for the mechanical PR interval: 122.4 ± 10.3 ms. On the other side, this study observed that mechanical PR was influenced by gestational age and fetal heart rate, independently; consequently, these variables should be evaluated in anti-Ro-exposed fetuses (34).

Up to the present time, data on the normal mechanical PR interval and its link both to gestational age and fetal heart rate are well-described by Phoon et al. (35).

As regards the different methods, Andelfinger et al. by evaluating the usefulness of the MV-Ao and SVC-Ao methods, showed no differences between the two methods (36). Moreover, Friedman et al. in their review concluded that both methods are widely feasible to the practitioner, with a normal range of PR interval equal to 100–135 ms (37).

When anti-Ro/SSA-exposed fetuses are considered, MV-Ao PR intervals were validated with either fetal magnetocardiography or postnatal ECHO (38). Using the method of MV-Ao PR intervals, a cut off of 150 ms was reported by different studies as too long PR interval (31, 37, 38). In conclusion, a MV-Ao PR interval ≥150 ms is “highly suggestive” and ≥160 ms “diagnostic” of fetal first-degree block (35, 38).

Could mechanical PR interval be classified as a biomarker for CHB? This was the aim of some studies investigating anti-Ro/SSA and anti-La/SSB pregnancies in order to early diagnose fetal anomalies and establish successive treatment. In 2004, Sonesson et al. evaluated 24 anti-Ro/SSA-exposed pregnancies between 18 and 24 weeks of gestation by weekly ECHO, defining a first-degree CHB by the presence of two consecutive AV interval measurements >95% percentiles in comparison to their controls. They detected signs of first-degree block in eight fetuses (30%) (39). One of these transitioned to CCHB; another case had rescue from second-degree to first-degree block after treatment with betamethasone, but in the other six cases normal sinus rhythm was restored before or after birth without any treatment (39). Thus, we should take into account that diagnose of the first-degree block is a variable condition, that has to be monitored and evaluated along time.

Subsequently, the PRIDE (PR Interval and Dexamethasone Evaluation) study, evaluating anti-Ro/SSA-exposed pregnancies (*n* = 98), found signs of first-degree block in 3% of cases (37). Furthermore, among three cases of third-degree block identified in the study, none was preceded by a first or second CHB; none was reversed by the maternal treatment. In this study, the tissue injury was described to be so rapid as to pass unnoticed by weekly ECHO (37). Thus, third-degree block can occur within <1 week from a normal rhythm.

Similar results were observed in a prospective study of 212 anti-Ro52-exposed pregnancies, highlighting that PR interval poorly predicted the CHB progression; nevertheless, the authors of this study suggested that ECHO monitoring remains useful to identify fetuses with second or third-degree block. In these cases an early treatment could improve fetal outcome (40).

It should be mentioned that a prolongation of PR interval can be spontaneously reversible, due to vagal tone, drugs, or transient damage, or it may be permanent and evolve to definite injury of the conduction cardiac tissue (41). Consequently, the identification of the first-degree is an alarm bell for the clinicians since it can progress (41).

Friedman et al. reported similar observations for the first-degree block; they recommend treatment with fluorinated steroids starting from the second-degree block and indicated the same treatment also in acute third-degree block, considering the rare possibility of reversibility (42).

Therefore, on the basis of the rapid transition from normal rhythm to advanced block within 1 week and its occurrence time before 24 weeks of gestation, it would be indicated to perform weekly ECHO surveillance almost between 16 and 24 weeks (41).

More recently, Cuneo et al. suggested that home monitoring of fetal heart rhythm (FHRM) is a feasible and reassuring method to surveillance anti-Ro-exposed pregnancies (43). In this study, the mothers using a commercially home Doppler device monitored twice-daily fetal heart rate. When fetal heart rate was detected as abnormal, a diagnostic ECHO was performed within few hours. The authors suggested that FHRM resulted a useful guide for early detection of CHB and the women were accomplished with this technique.

In conclusions, a PR interval prolongation above 150 ms by ECHO monitoring can be considered a useful “biomarker” of reversible injury, so ECHO monitoring has to be at least weekly performed in all anti-Ro-exposed pregnancies from 16 to at least 24 weeks of gestation. Furthermore, promising results seem to be achievable by home FHRM combined to ECHO monitoring. The early detection of conduction abnormalities can help the clinicians to identify the window of treatment. Fluorinated steroids are recommended in second-degree block and in acute third-degree block, considering the possibility of reversibility.

### Pharmacological Prevention of CHB During Pregnancy

Concerning the possibility of pharmacological prevention of CHB during pregnancy, efficacy of IVIG and/or fluorinated steroids throughout pregnancy have not been demonstrated.

To date, only maternal administration of Hydroxychloroquine (HCQ) has been shown to have an important role in preventing CHB. HCQ can act by different mechanisms as modulating the action of toll-like receptor and autoantibody-dependent type I IFN activation on the fetus.

HCQ can be employed both during pregnancy and breastfeeding, because small amount of the drug was observed in breast milk. Probably, adverse effects of HCQ could be on the QT interval (3), particularly when HCQ is associated with other QT-prolonging drugs, such as ondansetron, azithromycin, and oxytocin. In addition, women during pregnancy can have low magnesium, calcium, and 25-hydroxy vitamin D levels, which can increase this risk (3).

The HCQ effects on the QT of fetus before or with CHB are unknown (3). In CHB, many fetuses will demonstrate an excessive QT prolongation. Among antenatal diagnostic tools, only fetal magnetocardiography is currently approved by the Food and Drug Administration and is capable of measuring the QT; therefore, it may be performed in pregnancies treated with HCQ (44).

Izmirly et al. in a retrospective study firstly highlighted that HCQ significantly decreased the occurrence of CHB in patients having a previous affected child (45, 46).

More recently, Izmirly et al. (8) published a multicentre, open-label, single-arm, 2-stage clinical trial enrolling anti-Ro-exposed mothers at risk for CHB recurrence. They demonstrated that the early treatment with HCQ (at a dosage of 400 mg daily) decreased the recurrence of >50%. Since drug half-life is almost equal to 2 months, they recommend giving HCQ by completion of 10 weeks' gestation to obtain the best effect during the timing of CHB development (18–25 weeks of gestational age). Thus, HCQ is strongly indicated for prophylaxis in pregnant women with previous child affected by CHB. To date, this prophylaxis is not still recommended in the low-risk patients, such as anti-Ro/SSA positive women without any previous affected child. It could be estimated that, given a 50% decrease, it would be required to treat 100 low risk cases to prevent one case of CHB.

When first-degree heart block is seen on fetal ECHO, there is not a wide consensus as to the administration of drugs, as fluorinated steroids or HCQ, or no medication at all (47). The personal recommendations by Dr Jill Buyon is to start dexamethasone 4 mg a day for 1 week, and when deterioration to third degree is detected, dexamethasone could be discontinued (47). If the first-degree block reverses to normal rhythm or remains stable, it is difficult to establish if steroid treatment should be continued or not (47).

Future studies will clarify if the condition of first-degree block needs maternal treatment, and how long it will be done.

However, the physicians can obviously use HCQ in pregnant women with active lupus or other autoimmune disease or high anti-Ro/SSA titres also in absence of *prior* affected child: the HCQ treatment in these cases is primarily given for mother health.

## Conclusions

The identification of accurate biomarkers of women at risk to deliver a child with autoimmune CHB is still challenging.

Biochemical maternal tests could be used as susceptibility/risk biomarker of CHB, while the prolongation of the PR interval by ECHOs could represent a “monitoring” and “diagnostic” biomarker of CHB (48). In fact, the new knowledge indicates that maternal IFN signature and maternal antibodies profile, such as the combination of anti-Ro52 and anti-Ro60 antibodies positivity, the positivity of anti-p200 antibodies, particularly at high levels, can identify women at higher risk for delivering child with CHB. On the other hand, the prolongation of the PR interval by ECHOs represents is an indispensable tool for the diagnosis of fetal cardiac tissue injury and for maternal treatment timing.

Home monitoring of fetal heart rhythm (FHRM) is a feasible and reassuring method to surveil anti-Ro-exposed pregnancies and can be considered another novel monitoring biomarker for CHB.

Finally, the usefulness of maternal prophylaxis with HCQ is a very important key message, and is actually recommended in all women at risk for recurrence of CHB.

## Author Contributions

SDC and MPDC: study conception and design. SDC, CG, EG, AB, SS, and ST: acquisition and analysis of data. MPDC, SDC, CG, EG, ST, SS, and AB: interpretation of data. SDC, CG, MPDC, AB, EG, ST, and SS: original draft preparation. SDC, CG, MPDC, AB, ST, SS, and EG: writing, review and editing.

## Conflict of Interest

The authors declare that the research was conducted in the absence of any commercial or financial relationships that could be construed as a potential conflict of interest.
